# A Review of the Classical Canonical Ensemble Treatment of Newton’s Gravitation

**DOI:** 10.3390/e21070677

**Published:** 2019-07-11

**Authors:** Flavia Pennini, Angel Plastino, Mario Rocca, Gustavo Ferri

**Affiliations:** 1 Fac. de C. Exactas-National University La Pampa, Peru y Uruguay, Santa Rosa, La Pampa 6300, Argentina; 2 Consejo Nacional de Investigaciones Científicas y Tecnológicas, (IFLP-CCT-CONICET)-C. C. 727, 1900 La Plata, Argentina; 3 Departamento de Física, Universidad Católica del Norte, Av. Angamos 0610, Antofagasta 2340000, Chile; 4 Departamento de Física, Universidad Nacional de La Plata, La Plata 1900, Argentina; 5 Departamento de Matemática, Universidad Nacional de La Plata, La Plata 1900, Argentina

**Keywords:** Boltzmann-Gibbs distribution, divergences, dimensional regularization, specific heat

## Abstract

It is common lore that the canonical gravitational partition function Z associated with the classical Boltzmann-Gibbs (BG) exponential distribution cannot be built up because of mathematical pitfalls. The integral needed for writing up Z diverges. We review here how to avoid this pitfall and obtain a (classical) statistical mechanics of Newton’s gravitation. This is done using (1) the analytical extension treatment obtained of Gradshteyn and Rizhik and (2) the well known dimensional regularization technique.

## 1. Introduction

Common lore asserts that, in dealing with gravity, the classical Boltzmann-Gibbs (BG) probability distribution is unable to produce finite results. Why? Because the pertinent partition function Z diverges in any ν dimensional space [1,2,3,4,5]. If one calls *m* and *M* the masses involved, ν the number of space-dimensions, *G* the gravitation constant, β the inverse temperature, and *x*-*p* the phase-space coordinates, one has

(1) $$Z_{\nu }=\int_{R}e^{-\beta \left(\frac{p^{2}}{2m}-\frac{GmM}{r}\right)}d^{\nu }xd^{\nu }p,$$

involving a *positive* exponential. *However, common lore is not able to envision the possibility of taking care of such divergences via dimensional regularization*. Note that *Z* is not an observable, what matters is the convergence of physical expectations values one builds with it.

Dimensional regularization (DR) [6,7,8,9,10] can be regarded as a very important advance in theoretical physics. Actually DR is now well over 40 years old. It is appealed to in variegated branches of physics (see, as examples, the 54 references cited in Reference [11]).

The contents of Reference [12] will be reviewed here. There, for first time ever, a finite gravitational Z was computed for Boltzmann entropy using a generalization of the DR technique [12]. The feat was repeated, this time for Tsallis statistics in Reference [11]. We wish here to review these results in a rather accessible manner.

The present work intends to review the conjoining of gravitation plus DR to produce a finite thermo-statistics. The statistical mechanics of gravitation needs revisiting, we feel, because it has not yet percolated into the Statistical Mechanics community. Thus, in this effort we review the main points of how to overcome the above-mentioned technical problem of divergences by judicious use of an appropriate combination of (i) dimensional regularization and (ii) analytical extension. We remark that we deal only with two-body gravitational interactions. The more general *N*-bodies gravitational problem has not yet been solved. Indeed, it constitutes a frontier problem in Celestial Mechanics. We are not concerned with the general problem here.

Note also that, as is well known, at the *quantum field theory* level, the usual dimensional regularization cannot cope with the gravitational field (because it is non-renormalizable). Our present elaborations are of a quite different kind, though, because we will deal with Newton’s classical gravity. We apply our generalization of dimensional regularization [10], with which *it is possible treat non-renormalizable theories*.

## 2. Illustrating the Mechanism of Dimensional Regularization (DR) in a Simple Context

Let us discuss DR in a simple environment, accepting its justification as reported in Reference [10], that in turn generalizes References [6,7,8].

A first example is obtained using Tsallis’ *q*-entropy $S_{T}$[13] (q∈R). We look at a harmonic oscillator (HO) whose Hamiltonian reads simply

(2) $$H=P^{2}+Q^{2}.$$

Now, for this system the *q*-probability distribution $P_{t}$ can be cast as [13]

(3) $$P_{t}=Z^{-1}{\left[1+\left(1-q\right)\beta \left(P^{2}+Q^{2}\right)\right]}^{\frac{1}{q-1}}.$$

In turn, the q-entropy $S_{T}$ becomes

(4) $$S_{T}=\frac{1-\int_{R}\;P_{t}^{q}d\mu }{q-1}.$$

We concentrate our efforts on the partition function Z for the HO in three dimensions. The first DR idea is to re-write Z in ν dimensions, not in 3.

(5) $$Z=\int {\left[1+\left(1-q\right)\beta \left(P^{2}+Q^{2}\right)\right]}^{\frac{1}{q-1}}d^{\nu }pd^{\nu }q,$$

where $P^{2}=p_{1}^{2}+p_{2}^{2}+\cdots p_{\nu }^{2}$ and $Q^{2}=q_{1}^{2}+q_{2}^{2}+\cdots q_{\nu }^{2}$. In dealing with these kind of integrals one employs hyper-spherical coordinates and has

(6) $$Z=\frac{2\pi^{\nu }}{\Gamma (\nu )}\int_{0}^{\infty }s^{2\nu -1}{\left[1+\left(1-q\right)\beta s^{2}\right]}^{\frac{1}{q-1}}ds,$$

where $s^{2}=P^{2}+Q^{2}$. With the replacement $s^{2}=x$ one finds

(7) $$Z=\frac{\pi^{\nu }}{\Gamma (\nu )}\int_{0}^{\infty }\frac{x^{\nu -1}}{{\left[1+\left(1-q\right)\beta x\right]}^{\frac{1}{1-q}}}dx.$$

This integral can be looked up in Reference [14].

(8) $$\frac{\pi^{\nu }}{\Gamma (\nu )}\int_{0}^{\infty }\frac{x^{\mu -1}}{{\left(1+\gamma x\right)}^{v}}dx=\gamma^{-\mu }B\left(\mu ,v-\mu \right),$$

where B(μ,v−μ) is the celebrated Euler Beta function. Matching (7) to (8) one discovers that μ=ν, $v=\frac{1}{1-q}$, γ=(1−q)β. Accordingly,

(9) $$Z=\frac{\pi^{\nu }}{\Gamma (\nu )}{\left[\beta \left(1-q\right)\right]}^{-\nu }B\left(\nu ,\frac{1}{1-q}-\nu \right),$$

where

(10) $$B\left(\nu ,\frac{1}{1-q}-\nu \right)=\frac{\Gamma \left(\nu \right)\Gamma \left(\frac{1}{1-q}-\nu \right)}{\Gamma \left(\frac{1}{1-q}\right)}.$$

Note that Γ(z) stands for the well known Gamma function of Euler. It displays poles at z=0,−1,−2,−3,…. (all negative integer numbers and also zero). (10) entails

(11) $$Z={\left[\frac{\pi }{\beta (1-q)}\right]}^{\nu }\frac{\Gamma \left(\frac{1}{1-q}-\nu \right)}{\Gamma \left(\frac{1}{1-q}\right)}.$$

Following now Reference [11], we set $q=\frac{2}{3}$ in (11). Since Γ(3)=2, we are led to

(12) $$Z=\frac{1}{2}{\left(\frac{3\pi }{\beta }\right)}^{\nu }\Gamma \left(3-\nu \right).$$

One notes that for ν=3, Z does diverge. We have generalized Bollini-Giambiagi’s DR approach by performing a Laurent-expansion (LE) of Z around ν=3 and selecting after this what one regards as the physical Z-result, i.e., the ν−3-independent term of the LE. We justify this procedure in Appendix A. We now proceed with the LE by defining

(13) $$f\left(\nu \right)={\left(\frac{3\pi }{\beta }\right)}^{\nu },$$

whose (specializing Laurent to Taylor) Taylor-expansion around ν=3 is

(14) $$f\left(\nu \right)={\left(\frac{3\pi }{\beta }\right)}^{3}\sum_{n=0}^{\infty }\ln^{n}\left(\frac{3\pi }{\beta }\right)\frac{{\left(\nu -3\right)}^{n}}{n!}.$$

LE reads

(15) $$\Gamma \left(3-\nu \right)=\frac{1}{3-\nu }-C+\sum_{m=1}^{\infty }c_{m}{\left(3-\nu \right)}^{m},$$

C being Euler’s constant [14]. Multiplying the two series we obtain (the $c_{m}$ above and $a_{m}$ below are constants)

(16) $$f\left(\nu \right)\Gamma \left(3-\nu \right)={\left(\frac{3\pi }{\beta }\right)}^{3}\frac{1}{3-\nu }-{\left(\frac{3\pi }{\beta }\right)}^{3}C-{\left(\frac{3\pi }{\beta }\right)}^{3}\ln\left(\frac{3\pi }{\beta }\right)+\sum_{m=1}^{\infty }a_{m}{\left(3-\nu \right)}^{m}.$$

Then, Z becomes

(17) $$Z=-\frac{1}{2}{\left(\frac{3\pi }{\beta }\right)}^{3}\left[C+\ln\left(\frac{3\pi }{\beta }\right)\right],$$

or,

(18) $$Z=-\frac{1}{2}{\left(3\pi k_{B}T\right)}^{3}\left[C+\ln\left(3\pi k_{B}T\right)\right].$$

Since we need Z>0 [since all thermal quantities are to be real and expressed in terms of lnZ], *T* has to obey

(19) $$0<T<\frac{e^{-C}}{3\pi k_{B}},$$

entailing an upper bound for *T*. We have seen above that DR is indeed an accessible procedure that produces a finite partition function. We will use it for the gravitational interaction below.

## 3. Tsallis Statistical Treatment of Gravity

Consider the two-body Newton gravity and its Tsallis’ statistical mechanics. *m* and *M* are the involved masses. *M* is assumed to be at rest at the origin, while *m* moves. *G* is the gravitation constant. With reference to the precedent Section we see that the partition function is

(20) $$Z_{\nu }=\int_{R}\;{\left[1+\left(1-q\right)\beta \left(\frac{p^{2}}{2m}-\frac{GmM}{r}\right)\right]}_{+}^{\frac{1}{q-1}}d^{\nu }xd^{\nu }p,$$

where with ${\left[\right]}_{+}$ we understand a positive bracket [one takes into account only that variables’ region where the bracket is positive]. This is called the Tsallis-cut off [13]. In the integration process one uses, as above, hyper-spherical coordinates and two integrals, each in ν dimensions. We are left with only two radial coordinates (one in spatial coordinates and the other in momentum ones), together with 2(ν−1) angles. As the argument of the brackets should be positive, we have

(21) $$Z\nu ={\left[\frac{2\pi^{\frac{\nu }{2}}}{\Gamma \left(\frac{\nu }{2}\right)}\right]}^{2}{\left[\beta \left(q-1\right)\right]}^{\frac{1}{q-1}}\int_{0}^{\infty }r^{\nu -1}dr\times \;\int_{0}^{\sqrt{2m\left(\frac{1}{\beta (q-1)}+\frac{GmM}{r}\right)}}p^{\nu -1}{\left[\frac{1}{\beta (q-1)}+\frac{GmM}{r}-\frac{p^{2}}{2m}\right]}^{\frac{1}{q-1}}dp.$$

Again, using ${\left[\right]}_{+}$ means that one takes into account only that *p* region where the bracket is positive, which entails that our *p* integration must run from 0 to $\sqrt{2m\left(\frac{1}{\beta (q-1)}+\frac{GmM}{r}\right)}$. Our two integrals can be computed by appeal to Euler’s Beta function B [14]. Consider the first integral, that we call $I_{1}$.

(22) $$I_{1}=\int_{0}^{\sqrt{2m\left(\frac{1}{\beta (q-1)}+\frac{GmM}{r}\right)}}p^{\nu -1}{\left[\frac{1}{\beta (q-1)}+\frac{GmM}{r}-\frac{p^{2}}{2m}\right]}^{\frac{1}{q-1}}dp=\;{\left[\frac{1}{\beta (q-1)}+\frac{GmM}{r}\right]}^{\frac{\nu }{2}+\frac{1}{q-1}}B\left(\frac{\nu }{2},\frac{1}{q-1}+1\right).$$

Thus,

(23) $$Z_{\nu }=\frac{2\pi^{\nu }{\left(2m\right)}^{\frac{\nu }{2}}}{{\left[\Gamma \left(\frac{\nu }{2}\right)\right]}^{2}}{\left[\beta \left(q-1\right)\right]}^{\frac{\nu }{2}}{\left(GmM\right)}^{\nu }B\left(\frac{\nu }{2},\frac{1}{q-1}+1\right)B\left(\frac{\nu }{2}+\frac{1}{1-q},-\nu \right).$$

We see that poles emerge at any (entire) dimension ν, ν=3 included. We do need to appeal to dimensional regularization (DR). We use the DR-generalization given in Reference [10]

We then face, with M the pertinent integration-domain in our ν-phase space,

$$Z_{\nu }{\langle U\rangle }_{\nu }=\int_{M}{\left[1+\left(1-q\right)\beta \left(\frac{p^{2}}{2m}-\frac{GmM}{r}\right)\right]}_{+}^{\frac{1}{q-1}}\left(\frac{p^{2}}{2m}-\frac{GmM}{r}\right)d^{\nu }xd^{\nu }p=\;{\left[\frac{2\pi^{\frac{\nu }{2}}}{\Gamma \left(\frac{\nu }{2}\right)}\right]}^{2}{\left[\beta \left(q-1\right)\right]}^{\frac{1}{q-1}}\left\{\int_{0}^{\infty }r^{\nu -1}\right.dr\times$$

(24) $$\int_{0}^{\sqrt{2m\left(\frac{1}{\beta (q-1)}+\frac{GmM}{r}\right)}}\frac{p^{\nu +1}}{2m}{\left[\frac{1}{\beta (q-1)}+\frac{GmM}{r}-\frac{p^{2}}{2m}\right]}^{\frac{1}{q-1}}dp-\;\left.GmM\int_{0}^{\infty }r^{\nu -2}dr\int_{0}^{\sqrt{2m\left(\frac{1}{\beta (q-1)}+\frac{GmM}{r}\right)}}p^{\nu -1}{\left[\frac{1}{\beta (q-1)}+\frac{GmM}{r}-\frac{p^{2}}{2m}\right]}^{\frac{1}{q-1}}dp\right\}.$$

We remind in passing that Beta functions were invented by Euler and they appear in almost all fields of physics. Via the Beta function one finds

(25) $${\langle U\rangle }_{\nu }=\frac{2\pi^{\nu }{\left(2m\right)}^{\frac{\nu }{2}}}{Z_{\nu }{\left[\Gamma \left(\frac{\nu }{2}\right)\right]}^{2}}{\left[\beta \left(q-1\right)\right]}^{\frac{\nu }{2}-1}{\left(GmM\right)}^{\nu }\left[B\left(\frac{\nu }{2}+1,\frac{1}{q-1}+1\right)\times \right.\;\left.B\left(\frac{\nu }{2}+\frac{1}{1-q},1-\nu \right)-B\left(\frac{\nu }{2},\frac{1}{q-1}+1\right)B\left(\frac{\nu }{2}+\frac{1}{1-q}-1,1-\nu \right)\right].$$

### 3.1. Specialization to the Three-Dimensional Tsallis’ Environment

Let us deal with the $q=\frac{3}{2}$ instance, as done in Reference [11]. We go back now to (23), and work out the DR method and its associated Laurent expansion. We have

(25) $$Z_{\nu }=-\frac{8\pi^{2}{\left(\beta G^{2}m^{3}M^{2}\right)}^{\frac{3}{2}}}{3(\nu -3)}+\frac{4\pi^{2}}{3}{\left(\beta G^{2}m^{3}M^{2}\right)}^{\frac{3}{2}}\left[\frac{23}{3}-\ln\left(16\pi^{2}\beta G^{2}m^{3}M^{2}\right)\right]+\;\sum_{s=1}^{\infty }a_{s}{\left(\nu -3\right)}^{s}.$$

Here, we need the constant term in this Laurent expansion, which provides the physical value of the expansion. Accordingly,

(27) $$Z=\frac{4\pi^{2}}{3}{\left(\beta G^{2}m^{3}M^{2}\right)}^{\frac{3}{2}}\left[\frac{23}{3}-\ln\left(16\pi^{2}\beta G^{2}m^{3}M^{2}\right)\right].$$

As Z should be positive, one encounters a temperature-lower bound

(28) $$T>\frac{e^{-\frac{23}{3}}}{k_{B}}16\pi^{2}G^{2}m^{3}M^{2}.$$

The temperature cannot fall below the indicated value. This is interesting because classical theories like ours are not valid at low temperatures and *the present* treatment seems to be aware of this well known fact.

Analogously, from (25), we obtain for 〈U〉

(29) $$Z_{\nu }{\langle U\rangle }_{\nu }=\frac{32\pi^{2}{\left(\beta G^{2}m^{3}M^{2}\right)}^{\frac{3}{2}}}{3(\nu -3)}-\frac{32\pi^{2}}{3}{\left(\beta G^{2}m^{3}M^{2}\right)}^{\frac{3}{2}}\left[3-\ln\left(\pi^{2}\beta G^{2}m^{3}M^{2}\right)\right]+\;\sum_{s=1}^{\infty }a_{s}{\left(\nu -3\right)}^{s},$$

so that, for ν=3,

(30) $$Z\langle U\rangle =\frac{32\pi^{2}}{\beta }{\left(\beta G^{2}m^{3}M^{2}\right)}^{\frac{3}{2}}\left[\ln\left(\pi^{2}\beta G^{2}m^{3}M^{2}\right)-3+2C\right],$$

and

(31) $$\langle U\rangle =\frac{8[\ln\left(\pi^{2}\beta G^{2}m^{3}M^{2}\right)-3+2C]}{\beta [\frac{23}{3}-\ln\left(16\pi^{2}\beta G^{2}m^{3}M^{2}\right)]}.$$

where C is the Euler’s constant [14]. We have succeeded and are in possession of finite thermodynamical quantities, derived from finite values of the partition function and the mean energy.

### 3.2. Predicted Specific Heat

We look now for a specific heat built up as $C=\frac{\partial \langle U\rangle }{\partial T}$. For $q=\frac{3}{2}$ we find

(32) $$C=\frac{8k[\ln\left(\pi^{2}G^{2}m^{3}M^{2}\right)-4-\ln\left(kT\right)+2C]}{\frac{22}{3}+\ln\left(kT\right)-\ln\left(16\pi^{2}G^{2}m^{3}M^{2}\right)}-\;\frac{8k[3\ln\left(\pi^{2}G^{2}m^{3}M^{2}\right)-3-\ln\left(kT\right)+2C]}{{\left[\frac{22}{3}+\ln\left(kT\right)-\ln\left(16\pi^{2}G^{2}m^{3}M^{2}\right)\right]}^{2}}$$

**Figure 1:**
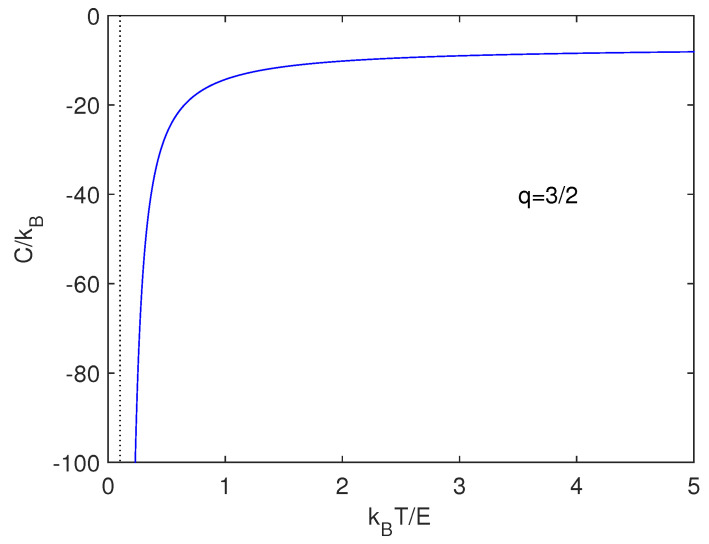
Specific heat versus $k_{B}T/E$ for q=3/2. We know that gravitational effects lead to negative specific heats [1]. This is clearly appreciated here.

Figure 1 depicts the specific heat corresponding to Equation (31). One defines $E=G^{2}m^{3}M^{2}$ with m≪M and express our quantities in $k_{B}T/E$-units. *The specific heat is negative*, as Astrophysics indicates for gravitation [1]. This property has been linked to self-gravitational systems [1].

## 4. Boltzmann-Gibbs Statistics

We pass now to the orthodox statistical formalism, which poses new challenges.

### 4.1. Analytic Extension

Analytic extension (or continuation) is a technique to extend the domain of a given analytic function. Analytic continuation often succeeds in defining further values of a function, for example in a new region where an infinite series representation in terms of which it is initially defined becomes divergent. Here we should keep in mind that one is dealing with the integral of an exponentially increasing function in βGmM/r given by (1).

We appeal in what follows to the use of analytical extension to obtain the result of some useful integrals that we will need later on. The discussion is rather technical and can be skipped without losing sight of our objectives. Whittaker functions play a protagonic role. Such functions are special solutions of Whittaker’s equation, a modified form of the confluent hypergeometric equation. Equations of motion for holonomic systems are Whittaker’s equations (system are called holonomic if all their constraints are holonomic, and a constraint is holonomic if it is expressible as a function).

### 4.2. Helpful Integrals

Recourse is now to be made to Table [14], where one finds a helpful integral for us, after specializing it. We speak of

(33) $$\int_{0}^{\infty }x^{\nu -1}{\left(x+\gamma \right)}^{\mu -1}e^{-\frac{\beta }{x}}dx=\beta^{\frac{\nu -1}{2}}\gamma^{\frac{\nu -1}{2}+\mu }\Gamma \left(1-\mu -\nu \right)e^{\frac{\beta }{2\gamma }}W_{\frac{\nu -1}{2}+\mu ,-\frac{\nu }{2}}\left(\frac{\beta }{\gamma }\right),$$

where |arg(γ)|<π. Also, ℜ(1−μ−ν)>0. *W* is *one* of the two Whittaker functions. Here we do not demand ℜβ>0, as emphasized by Gradshteyn and Rizhik [14] [see the picture in page 340, Equation (7), named ET II 234(13)a, with reference made to Reference [15] (Caltech’s Bateman Project)]. The final letter “a” stands for analytical extension. Choosing μ=1 one has

(34) $$\int_{0}^{\infty }x^{\nu -1}e^{-\frac{\beta }{x}}dx=\beta^{\frac{\nu -1}{2}}\gamma^{\frac{\nu +1}{2}}\Gamma \left(-\nu \right)e^{\frac{\beta }{2\gamma }}W_{\frac{\nu +1}{2},-\frac{\nu }{2}}\left(\frac{\beta }{\gamma }\right),$$

for ν≠0,−1,−2,−3,.…. In addition [14],

(35) $$W_{\frac{\nu +1}{2},-\frac{\nu }{2}}\left(\frac{\beta }{\gamma }\right)=M_{\frac{\nu +1}{2},\frac{\nu }{2}}\left(\frac{\beta }{\gamma }\right)={\left(\frac{\beta }{\gamma }\right)}^{\frac{\nu +1}{2}}e^{-\frac{\beta }{2\gamma }}.$$

Here. *M* is *Whittaker’s function two*. Thus,

(36) $$\int_{0}^{\infty }x^{\nu -1}e^{-\frac{\beta }{x}}dx=\beta^{\nu }\Gamma \left(-\nu \right),$$

which one can evaluate for all ν=1,2,3,…. by recourse to the DR approach [6,7,8,10]. If we turn β into −β in (33) we have to deal with

(37) $$\int_{0}^{\infty }x^{\nu -1}{\left(x+\gamma \right)}^{\mu -1}e^{\frac{\beta }{x}}dx={\left(-\beta \right)}^{\frac{\nu -1}{2}}\gamma^{\frac{\nu -1}{2}+\mu }\Gamma \left(1-\mu -\nu \right)e^{-\frac{\beta }{2\gamma }}W_{\frac{\nu -1}{2}+\mu ,-\frac{\nu }{2}}\left(-\frac{\beta }{\gamma }\right).$$

Again, one chooses μ=1 so that

(38) $$\int_{0}^{\infty }x^{\nu -1}e^{\frac{\beta }{x}}dx={\left(-\beta \right)}^{\frac{\nu -1}{2}}\gamma^{\frac{\nu +1}{2}}\Gamma \left(-\nu \right)e^{-\frac{\beta }{2\gamma }}W_{\frac{\nu +1}{2},-\frac{\nu }{2}}\left(-\frac{\beta }{\gamma }\right),$$

that holds for ν≠0,−1,−2,−3,……

We tackle now

(39) $$W_{\frac{\nu +1}{2},-\frac{\nu }{2}}\left(-\frac{\beta }{\gamma }\right)=M_{\frac{\nu +1}{2},\frac{\nu }{2}}\left(-\frac{\beta }{\gamma }\right)={\left(-\frac{\beta }{\gamma }\right)}^{\frac{\nu +1}{2}}e^{\frac{\beta }{2\gamma }},$$

plus

(40) $$\int_{0}^{\infty }x^{\nu -1}e^{\frac{\beta }{x}}dx={\left(-\beta \right)}^{\nu }\Gamma \left(-\nu \right),$$

equivalent to interchange β with −β in (36). We arrive at the fact that restricting the analytical extension (AE) of (33) is the same as effecting the AE of the restriction. This reaffirms that Gradshteyn and Rizhik’s AE is valid. Equation (40) exhibits a cut at ℜβ>0. We are entitled to select then ${\left(-\beta \right)}^{\nu }=e^{i\pi \nu }\beta^{\nu }$, ${\left(-\beta \right)}^{\nu }=e^{-i\pi \nu }\beta^{\nu }$, or ${\left(-\beta \right)}^{\nu }=\cos\left(\pi \nu \right)\beta^{\nu }$. We choose the latter possibility and find

(41) $$\int_{0}^{\infty }x^{\nu -1}e^{\frac{\beta }{x}}dx=\cos\left(\pi \nu \right)\beta^{\nu }\Gamma \left(-\nu \right),$$

a useful result, to be employed in what follows.

Reference [14] allows us to realize that

(42) $$\int_{0}^{\infty }x^{\nu -1}e^{-\beta x^{2}-\gamma x}dx={\left(2\beta \right)}^{-\frac{\nu }{2}}\Gamma \left(\nu \right)e^{\frac{\gamma^{2}}{8\beta }}D_{-\nu }\left(\frac{\gamma }{\sqrt{2\beta }}\right),$$

with *D* being the parabolic-cylinder function. Choosing γ=0 we encounter

(43) $$\int_{0}^{\infty }x^{\nu -1}e^{-\beta x^{2}}dx={\left(2\beta \right)}^{-\frac{\nu }{2}}\Gamma \left(\nu \right)D_{-\nu }\left(0\right).$$

Given that

(44) $$D_{-\nu }\left(0\right)=\frac{2^{-\frac{\nu }{2}}\sqrt{\pi }}{\Gamma \left(\frac{\nu +1}{2}\right)},$$

one has

(45) $$\int_{0}^{\infty }x^{\nu -1}e^{-\beta x^{2}}dx=\frac{2^{-\nu }\beta^{-\frac{\nu }{2}}\sqrt{\pi }\;\Gamma \left(\nu \right)}{\Gamma \left(\frac{\nu +1}{2}\right)},$$

still another helpful result for our future considerations.

## 5. The ν-Dimensional BG Distribution

The BG partition function $Z_{\nu }$ is well-known (see any text-book on Statistical Mechanics)

(46) $$Z_{\nu }=\int_{M}e^{-\beta \left(\frac{p^{2}}{2m}-\frac{GmM}{r}\right)}d^{\nu }xd^{\nu }p,$$

with β the inverse temperature, *m* and *M* the involved masses and *G* the gravitation constant. *M* is assumed to be at rest at the origin. In effecting the integration process one appeals to hyper-spherical coordinates for the two integrals, each in ν dimensions. The corresponding change of variables is

(47) $$x_{1}=r\cos\theta_{1}\;x_{2}=r\sin\theta_{1}\cos\theta_{2}\;x_{3}=r\sin\theta_{1}\sin\theta_{2}\cos\theta_{3}\;.\;.\;x_{\nu -1}=r\sin\theta_{1}\ldots \ldots \sin\theta_{\nu -2}\cos\theta_{\nu -1}\;x_{\nu }=\sin\theta_{1}\ldots \ldots \sin\theta_{\nu -1}\sin\theta_{\nu -1},$$

where $0\leq \theta_{j}\leq \pi$, 1≤j≤ν−2, and $0\leq \theta_{\nu -1}\leq 2\pi$. The integration on the angular variables ($\Omega_{\nu }=\left(\theta_{1},\theta_{2},\ldots ,\theta_{\nu -1}\right)$) yields as a result

(48) $$\int_{\Omega_{\nu }}d\Omega_{\nu }=\frac{2\pi^{\frac{\nu }{2}}}{\Gamma \left(\frac{\nu }{2}\right)}$$

We are left now with only two radial coordinates (one in *r*-space, the other in *p*-space). Then,

(49) $$Z_{\nu }={\left[\frac{2\pi^{\frac{\nu }{2}}}{\Gamma \left(\frac{\nu }{2}\right)}\right]}^{2}\overset{\infty }{\underset{0}{\;\int \int \;}}{\left(rp\right)}^{\nu -1}e^{-\beta \left(\frac{p^{2}}{2m}-\frac{GmM}{r}\right)}dr\;dp.$$

At this stage we need (41). Accordingly, $\int_{0}^{\infty }r^{\nu -1}e^{\beta \frac{GmM}{r}}dr$ and (45) for $\int_{0}^{\infty }p^{\nu -1}e^{-\beta \frac{p^{2}}{2m}}dp,$ so that we obtain

(50) $$Z_{\nu }=4\sqrt{\pi }\cos\left(\pi \nu \right){\left(\frac{\pi^{2}\beta G^{2}m^{3}M^{2}}{2}\right)}^{\frac{\nu }{2}}\frac{\Gamma (\nu )\Gamma (-\nu )}{{\left[\Gamma \left(\frac{\nu }{2}\right)\right]}^{2}\Gamma \left(\frac{\nu +1}{2}\right)}.$$

Looking at (50) one ascertains that poles emerge at any (entire) dimension ν, ν=3 included. Then, appeal to dimensional regularization (DR) to deal with them becomes mandatory. For this we used in Section 4 the generalized DR approach advanced in Reference [10].

Still, we need first an expression for the mean energy

(51) $${\langle U\rangle }_{\nu }=\frac{1}{Z_{\nu }}\int_{R}e^{-\beta \left(\frac{p^{2}}{2m}-\frac{GmM}{r}\right)}\left(\frac{p^{2}}{2m}-\frac{GmM}{r}\right)d^{\nu }xd^{\nu }p.$$

Invoking the hyper-spherical coordinates previously presented we find for ${\langle U\rangle }_{\nu }$

(52) $${\langle U\rangle }_{\nu }=\frac{1}{Z_{\nu }}{\left[\frac{2\pi^{\frac{\nu }{2}}}{\Gamma \left(\frac{\nu }{2}\right)}\right]}^{2}\overset{\infty }{\underset{o}{\;\int \int \;}}e^{-\beta \left(\frac{p^{2}}{2m}-\frac{GmM}{r}\right)}\left(\frac{p^{2}}{2m}-\frac{GmM}{r}\right)p^{\nu -1}r^{\nu -1}dp\;dr.$$

Now, we use again (41) and (45), which gives for the mean energy

(53) $${\langle U\rangle }_{\nu }=\frac{1}{Z_{\nu }}\frac{\sqrt{\pi }}{\beta }\cos\left(\pi \nu \right){\left(\frac{\pi^{2}\beta G^{2}m^{3}M^{2}}{2}\right)}^{\frac{\nu }{2}}\times \;\left[\frac{\Gamma (\nu +2)\Gamma (-\nu )}{{\left[\Gamma \left(\frac{\nu }{2}\right)\right]}^{2}\Gamma \left(\frac{\nu +3}{2}\right)}+4\frac{\Gamma (\nu )\Gamma (1-\nu )}{{\left[\Gamma \left(\frac{\nu }{2}\right)\right]}^{2}\Gamma \left(\frac{\nu +1}{2}\right)}\right].$$

In this Section, we have obtained the basic ingredients for working with the canonical ensemble. We now have the partition function for gravity and the mean energy. For them one can obtain the gravitational entropy, first of all, and all the relevant thermodynamic quantities associated with gravity.

### 5.1. The 3D Regularized BG Distribution

We go back to (50). The idea it to work the ensuing dimensional regularization (DR) process. If we have, for instance, an expression F(ν) that diverges, say, for ν=3, our Bollini-Giambiagi’s DR generalized approach consists in performing the Laurent-expansion of *F* around ν=3 and choose then, as the physical result for *F*, the ν=3-independent term in that expansion. The justification for this procedure is carefully explained in Reference [10] (see Appendix A).

In our present situation, the pertinent Laurent expansion in ν (around ν=3) takes the appearance

(54) $$Z_{\nu }=-\frac{1}{3\sqrt{\pi }}\left[1+\frac{2}{3(\nu -3)}\right]{\left(2\pi^{2}\beta G^{2}m^{3}M^{2}\right)}^{\frac{3}{2}}\times \;\left[\ln\left(2\pi^{2}\beta G^{2}m^{3}M^{2}\right)+C-\frac{17}{3}\right]+\sum_{s=1}^{\infty }a_{s}{\left(\nu -3\right)}^{s},$$

C standing for Euler’s constant. We see that $Z_{\nu }$ does diverge at ν=3. By fiat (and this is the central tenet of DR), the (ν−3)-independent term in the Laurent expansion of $Z_{\nu }$’s gives the physical value of the Z. Accordingly, from the ν-independent term one finds

(55) $$Z=\frac{1}{3\sqrt{\pi }}{\left(2\pi^{2}\beta G^{2}m^{3}M^{2}\right)}^{\frac{3}{2}}\left[\frac{17}{3}-C-\ln\left(8\pi^{2}\beta G^{2}m^{3}M^{2}\right)\right].$$

Since Z is necessarily positive, one encounters a temperature-lower bound

(56) $$T>\frac{e^{-\frac{17}{3}+C}}{k_{B}}8\pi^{2}G^{2}m^{3}M^{2},$$

than can not be perforated.

Analogously, from (53), one obtains for 〈U〉 the Laurent expansion

(57) $$Z{\langle U\rangle }_{\nu }=\frac{8}{\sqrt{\pi }\beta \left(\nu -3\right)}{\left(\frac{\pi^{2}\beta G^{2}m^{3}M^{2}}{2}\right)}^{\frac{3}{2}}+\frac{8}{\sqrt{\pi }\beta }{\left(\frac{\pi^{2}\beta G^{2}m^{3}M^{2}}{2}\right)}^{\frac{3}{2}}\times \;\left[\frac{1}{2}\ln\left(\frac{\pi^{2}\beta G^{2}m^{3}M^{2}}{2}\right)+2\ln2+\frac{C}{2}-\frac{5}{2}\right]+\sum_{s=1}^{\infty }a_{s}{\left(\nu -3\right)}^{s},$$

where Z is that of Equation (55). As above, the (ν−3)-independent term is the physical value of the mean energy 〈U〉

(58) $$\langle U\rangle =\frac{1}{Z}\frac{8}{\sqrt{\pi }\beta }{\left(\frac{\pi^{2}\beta G^{2}m^{3}M^{2}}{2}\right)}^{\frac{3}{2}}\left[\frac{1}{2}\ln\left(\frac{\pi^{2}\beta G^{2}m^{3}M^{2}}{2}\right)+2\ln2+\frac{C}{2}-\frac{5}{2}\right].$$

Replacing here Z’s physical value provided by Equation (55) we can write

(59) $$\langle U\rangle =-\frac{3}{2\beta }\frac{\ln\left(\pi^{2}\beta G^{2}m^{3}M^{2}\right)+3\ln2+C-5}{\ln\left(\pi^{2}\beta G^{2}m^{3}M^{2}\right)+\ln8+C-\frac{17}{3}}.$$

We have thus obtained finite gravitational values for the partition function and the mean energy. From them, we get of course all remaining thermodynamic quantities.

### 5.2. BG Specific Heat

In Reference [12], the authors derived a canonical gravitational mean energy function. It is natural to now use it for computing the specific heat $C=\frac{\partial \langle U\rangle }{\partial T}$. This entails dealing with

(60) $$C=\frac{3k}{2}\frac{\ln(\pi^{2}\beta G^{2}m^{3}M^{2})+3\ln2-6+C}{\frac{17}{3}-C-\ln\left(2\pi^{2}\beta G^{2}m^{3}M^{2}\right)-\ln2}-\;\frac{3k}{2}\frac{\ln(16\pi^{2}\beta G^{2}m^{3}M^{2})+3\ln2-5+C}{{\left[\frac{17}{3}-C-\ln\left(2\pi^{2}\beta G^{2}m^{3}M^{2}\right)-\ln2\right]}^{2}}.$$

**Figure 2:**
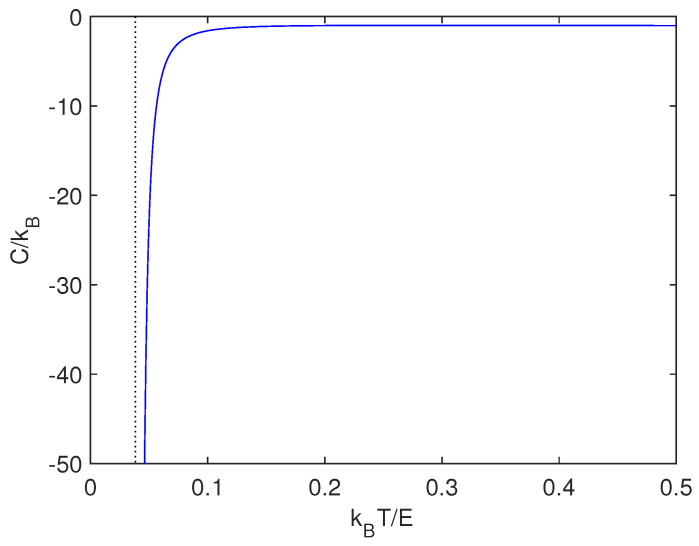
Specific heat versus $k_{B}T/E$.

Figure 2 displays C as given by Equation (60). Let us call $E=G^{2}m^{3}M^{2}$, with m≪M and express our quantities in $k_{B}T/E$-units. C turns out to be negative, as astrophysicists expect for gravitation [1]. Certainly, in astrophysics this fact is associated with self-gravitating systems [1]. Thirring did magnificently illustrate negative heat capacities [16,17]. In another vein, Verlinde has linked this sort of system with an entropic force [18]. It should seem natural to then guess that such an entropic force may appear at the energy-associated poles discussed above.

## 6. Conclusions

It is a common belief that the gravitational partition function Z linked to a Boltzmann-Gibbs (BG) probability distribution does diverge [1,2].

Here, we tried to undermine this belief as false and circumvented the problem by contemplating the possibility of dimensional regularization. Analytical extension plus dimensional regularization (DR) allows one to encounter finite Z (see Appendix A).

In preparation for dealing with the Boltzmann-Gibbs approach we worked out the pertinent, easier Tsallis problem and investigated the poles both in its Tsallis’ Z and in the average energy. These poles emerge at specific, discrete q-values. We studied the thermodynamic behavior at the poles and encountered notable peculiarities. Having succeeded in this Tsallis case, we tackled the orthodox Boltzmann-Gibbs (BG) case and were able to successfully remove the emerging singularities.

The treatment of the gravitational problem is considerably more difficult in the BG than in the Tsallis scenario. The latter only requires dimensional regularization. For BG we need analytic extension as well.

It is important to emphasize that our generalized DR treatment of Reference [10], not the original DR formulation, is the treatment that allows one to bypass divergences in the canonical ensemble Newtonian Z.

## Appendix A. Our Generalization of the Dimensional Regularization Technique

QFT inherits the problem of defining the product of two distributions (PTD) (a product in a ring with divisors of zero), an old conundrum of functional analysis. This is because in QFT the problem of evaluating the product of distributions with coincident point singularities is intimately linked to the asymptotic behaviour of loop integrals.

From a mathematical point of view, almost all PTD-definitions lead to limitations on the set of distributions that can be multiplied by each other to yield another distribution of the same kind. In fact, the great mathematician Laurent Schwartz himself was not able to define a product of distributions regarded as an algebra, instead of as a ring with divisors of zero.

References [19,20,21,22] showed that it is possible to define a general convolution between the ultradistributions of the portuguese mathematician J. Sebastiao e Silva (JSS) [23] (called Ultrahyperfunctions). Such a convolution produces another ultrahyperfunction. Therefore, we face a product in a ring with zero divisors. Such a ring is the space of distributions of exponential type, or ultradistributions of exponential type, obtained applying an anti-Fourier transform to the space of tempered ultradistributions or ultradistributions of exponential kind. Remark that the ultrahyperfunctions are the generalization and extension to the complex plane of the Schwartz tempered distributions and the distributions of exponential type. That is, the temperate distributions and those of exponential type are a subset of the ultrahyprefunctions. The problem we then face is that of formulating the convolution between ultradistributions. This is a complex issue, difficult to manage. Fortunately, we have found that a method similar to that used to define the convolution of ultradistributions can also be used to define the convolution of Lorentz Invariant distributions using the dimensional regularization approach (DR) in momentum space.

As a consequence, ultradistributions need not to be used in the calculations we need to do here, which considerably simplifies them. Taking advantage of our DR-treatment one can also work in configuration space [8]. Thus, one can obtain a convolution of Lorentz invariant tempered distributions in momentum space and the corresponding product in configuration space. Technically, our DR generalization happens to be a convolution of distributions called STDELI (see the main text of the paper) in momentum space and a product in a ring with divisors of zero in configuration space. These STDELI distributions have nice properties. In particular, we are guaranteed that our distribution-products both exist and are finite.

In DR one basically Laurent-expands the quantity to be regularized in terms of the dimension (regarded as a real number) and keeps just that special term of the expansion that does not depend on the dimension. What is the importance of using only *that term independent of the dimension?* That the result obtained for finite convolutions will coincide with that term. This translates to configuration space the product-operation in a ring with divisors of zero.

### Appendix A.1. Lorentz Invariant Tempered Distributions

This subsection provides the definitions that we will use in this appendix. We consider first the case on the ν-dimensional Minkowskian space $M_{\nu }$. Let $S^{{}^{\prime }}$ be the space of Schwartz tempered distributions [24,25]. Let $g\in S^{{}^{\prime }}$. We assert that $g\in S_{L}^{{}^{\prime }}$ if and only if

(A1) $$g\left(\rho \right)=\frac{d^{l}}{d\rho^{l}}f\left(\rho \right),$$

where the derivative is to be considered in what is called the sense of distributions, *l is a natural number*, $\rho =k^{2}=k_{0}^{2}-k_{1}^{2}-k_{2}^{2}-\cdots -k_{\nu -1}^{2}$, and *f* satisfies

(A2) $$\int_{-\infty }^{\infty }\frac{\left|f(\rho )\right|}{{\left(1+\rho^{2}\right)}^{n}}d\rho <\infty ,$$

being continuous in $M_{\nu }$. *The exponent n is a natural number.* We state then that $f\in T_{1L}$.

In the case of the Euclidean space $R_{\nu }$, let $g\in S^{{}^{\prime }}$. We assert that $g\in S_{R}^{{}^{\prime }}$ if and only if

(A3) $$g\left(k\right)=\frac{d^{l}}{dk^{l}}f\left(k\right),$$

where $k^{2}=k_{0}^{2}+k_{1}^{2}+k_{2}^{2}-\cdots +k_{\nu -1}^{2},$ with f(k) satisfying

(A4) $$\int_{0}^{\infty }\frac{\left|f(k)\right|}{{\left(1+k^{2}\right)}^{n}}dk<\infty ,$$

and f(k) being continuous in $R_{\nu }$. We state then that $f\in T_{1R}$.

We call $S_{LA}^{{}^{\prime }}$ and $S_{RA}^{{}^{\prime }}$ the Fourier anti-transformed spaces of $S_{L}^{{}^{\prime }}$ and $S_{R}^{{}^{\prime }}$, respectively.

### Appendix A.2. The Generalization of Dimensional Regularization from Configuration Space to Euclidean Space

The expression for the convolution of two spherically symmetric functions was deduced in Reference [8] (h(k,ν)=(f∗g)(k,ν)). One has

(A5) $$h\left(k,\nu \right)=\frac{2^{4-\nu }\pi^{\frac{\nu -1}{2}}}{\Gamma \left(\frac{\nu -1}{2}\right)k^{\nu -2}}\overset{\;\;\;\infty }{\underset{\;0}{\;\int \int \;}}f\left(k_{1},\nu \right)g\left(k_{2},\nu \right)\;\times \;{\left[4k_{1}^{2}k_{2}^{2}-{\left(k^{2}-k_{1}^{2}-k_{2}^{2}\right)}^{2}\right]}_{+}^{\frac{\nu -3}{2}}k_{1}k_{2}\;dk_{1}\;dk_{2}.$$

However, Bollini and Giambiagi were not able to not obtain a product in a ring with divisors of zero. This is something that we will do now. Consider here that *f* and *g* belong to $S_{R}^{{}^{\prime }}$. With the change of variables $\rho =k^{2}$, $\rho_{1}=k_{1}^{2}$, $\rho_{2}=k_{2}^{2}$ we have

(A6) $$h\left(\rho ,\nu \right)=\frac{2^{2-\nu }\pi^{\frac{\nu -1}{2}}}{\Gamma \left(\frac{\nu -1}{2}\right)\rho^{\frac{\nu -2}{2}}}\overset{\;\;\;\infty }{\underset{\;0}{\;\int \int \;}}f\left(\rho_{1},\nu \right)g\left(\rho_{2},\nu \right)\;\times \;{\left[4\rho_{1}\rho_{2}-{\left(\rho -\rho_{1}-\rho_{2}\right)}^{2}\right]}_{+}^{\frac{\nu -3}{2}}\;d\rho_{1}\;d\rho_{2}.$$

Let  be a vertical band contained in the complex ν-plane . Integral (A6) is an analytic function of ν defined in the domain . Then, according to the method of Reference [19], h(ν,ρ) can be analytically continued to other parts of . In particular, near the dimension $\nu_{0}$ we have Laurent’s expansion

(A7) $$h\left(\rho ,\nu \right)=\sum_{m=-1}^{\infty }h^{\left(m\right)}\left(\rho \right){\left(\nu -\nu_{0}\right)}^{m}.$$

Here, $\nu_{0}$ is the dimension of the space on which our interest is focused. In particular, $\nu_{0}=4$ is the dimension that we will consider here. We now *define the convolution-product* as the $\left(\nu -\nu_{0}\right)$-independent term of Laurent’s expansion, since the terms with m>0 vanish and the one with m=−1 displays a pole in (A7). Thus, it is natural to define

(A8) $$h_{\nu_{0}}\left(\rho \right)=h^{\left(0\right)}\left(\rho \right).$$

Let us insist: if the convolution is finite, the Laurent expansion reads

(A9) $$h\left(\rho ,\nu \right)=\sum_{m=0}^{\infty }h^{\left(m\right)}\left(\rho \right){\left(\nu -\nu_{0}\right)}^{m},$$

and the term $h^{\left(-1\right)}\left(\rho \right)$ is null. Therefore, in this case the value of the series in $\nu =\nu_{0}$ is precisely $h_{\nu_{0}}\left(\rho \right)=h^{\left(0\right)}\left(\rho \right)$. One thus *defines the convolution-product* as the $\left(\nu -\nu_{0}\right)$-independent term of the pertinent Laurent expansion.to obtain a product in a ring with zero divisors, as done in References [10,19,20,21,22]. Accordingly, in the ring with zero divisors $S_{RA}^{{}^{\prime }}$, we have defined a product of distributions in the manner above indicated.

### Appendix A.3. Example

As an example of the use of (A6) and (A8) we evaluate the convolution of a massless propagator with a propagator corresponding to a scalar particle of mass *m*.

(A10) $$\frac{1}{k^{2}}*\frac{1}{k^{2}+m^{2}}=\int \frac{d^{4}p}{p^{2}\left[{\left(k-p\right)}^{2}+m^{2}\right]}.$$

Generalizing this integral to ν dimensions we have

(A11) $${\left(\frac{1}{k^{2}}*\frac{1}{k^{2}+m^{2}}\right)}_{\nu }=\int \frac{d^{\nu }p}{p^{2}\left[{\left(k-p\right)}^{2}+m^{2}\right]}.$$

The result of this convolution is given in Reference [8]. The result reads

(A12) $$h\left(k,\nu \right)=2^{\nu -2}\pi^{\frac{\nu }{2}}m^{\nu -4}\frac{\Gamma \left(\frac{\nu -2}{2}\right)\Gamma \left(\frac{4-\nu }{2}\right)}{\Gamma \left(\frac{\nu }{2}\right)}F\left(1,\frac{4-\nu }{2};\frac{\nu }{2};-\frac{k^{2}}{m^{2}}\right).$$

Now we use the equality

(A13) $$\Gamma \left(\frac{4-\nu }{2}\right)F\left(1,\frac{4-\nu }{2};\frac{\nu }{2};-\frac{k^{2}}{m^{2}}\right)=\;\Gamma \left(\frac{4-\nu }{2}\right)-\frac{2}{\nu }\Gamma \left(\frac{6-\nu }{2}\right)\frac{k^{2}}{m^{2}}F\left(1,\frac{6-\nu }{2};\frac{2+\nu }{2};-\frac{k^{2}}{m^{2}}\right).$$

After a tedious calculation, we obtain the corresponding Laurent’s expansion of h(k,ν)

(A14) $$h\left(k,\nu \right)=-\frac{8\pi^{2}}{\nu -4}+4\pi^{2}\left(C+2-\ln4-\ln\pi -\ln m^{2}\right)-\;2\pi^{2}\frac{k^{2}}{m^{2}}F\left(1,1;3;-\frac{k^{2}}{m^{2}}\right)+\sum_{s=1}^{\infty }a_{s}{\left(\nu -4\right)}^{s},$$

where C is Euler’s constant with a changed sign C=−0.57721566490. Thus, we have

(A15) $$\frac{1}{k^{2}}*\frac{1}{k^{2}+m^{2}}=4\pi^{2}\left(C+2-\ln4-\ln\pi -\ln m^{2}\right)-\;2\pi^{2}\frac{k^{2}}{m^{2}}F\left(1,1;3;-\frac{k^{2}}{m^{2}}\right).$$

### Appendix A.4. The Generalization of Dimensional Regularization in Configuration Space to Minkowskian Space

In this section we repeat the efforts of the preceding one for Minkowskian space. The generalization of the so-called Bochner formula to Minkowskian space has been obtained in Reference [20]. Remember that ν indicates the dimensionality. The corresponding expression, for ν even, reads

(A16) $$h\left(\rho ,\nu \right)=\frac{\pi^{\frac{\nu -3}{2}}}{2^{\nu -1}}e^{\frac{i\pi (2-\nu )}{2}}\Gamma \left(\frac{3-\nu }{2}\right)\overset{\;\;\;\infty }{\underset{-\infty }{\;\int \int \;}}f\left(\rho_{1},\nu \right)g\left(\rho_{2},\nu \right)\;\;\;\times \;\left\{{\left(\rho -i0\right)}^{-\frac{1}{2}}{\left[\frac{{\left(\rho -\rho_{1}-\rho_{2}\right)}^{2}-4\rho_{1}\rho_{2}}{\rho }+i0\right]}^{\frac{\nu -3}{2}}+e^{i\pi (\nu -2)}\right.\;\;\;\times \;\left.{\left(\rho +i0\right)}^{-\frac{1}{2}}{\left[\frac{{\left(\rho -\rho_{1}-\rho_{2}\right)}^{2}-4\rho_{1}\rho_{2}}{\rho }-i0\right]}^{\frac{\nu -3}{2}}\right\}d\rho_{1}\;d\rho_{2}.$$

When ν is odd we find

(A17) $$h\left(\rho ,\nu \right)=-\frac{i\pi^{\frac{\nu -3}{2}}}{2^{\nu -1}\Gamma \left(\frac{\nu -3}{2}\right)}\overset{\;\;\;\infty }{\underset{-\infty }{\;\int \int \;}}f\left(\rho_{1},\nu \right)g\left(\rho_{2},\nu \right){\left[\frac{{\left(\rho -\rho_{1}-\rho_{2}\right)}^{2}-4\rho_{1}\rho_{2}}{\rho }\right]}^{\frac{\nu -3}{2}}\left\{{\left(\rho -i0\right)}^{-\frac{1}{2}}\right.\times \;\left[\psi \left(\frac{\nu -1}{2}\right)+\frac{i\pi }{2}+\ln\left[\frac{{\left(\rho -\rho_{1}-\rho_{2}\right)}^{2}-4\rho_{1}\rho_{2}}{\rho }+i0\right]\right]-{\left(\rho +i0\right)}^{-\frac{1}{2}}\;\left.\left[\psi \left(\frac{\nu -1}{2}\right)+\frac{i\pi }{2}+\ln\left[-\frac{{\left(\rho -\rho_{1}-\rho_{2}\right)}^{2}-4\rho_{1}\rho_{2}}{\rho }+i0\right]\right]\right\}d\rho_{1}\;d\rho_{2}.$$

For the Minkowskian case one can also employ Laurent’s expansion in the fashion

(A18) $$h\left(\rho ,\nu \right)=\sum_{m=-1}^{\infty }h^{\left(m\right)}\left(\rho \right){\left(\nu -\nu_{0}\right)}^{m},$$

and therefore, again, we have for the convolution the result

(A19) $$h_{\nu_{0}}\left(\rho \right)=h^{\left(0\right)}\left(\rho \right).$$

Thus, in the ring with zero divisors $S_{LA}^{{}^{\prime }}$ we have presented a product of distributions.

### Appendix A.5. Further Examples

As an example of the use of (A16) we will consider the convolution of two Dirac δ-distributions, δ(ρ). The result is

$$h\left(\rho ,\nu \right)=\frac{\pi^{\frac{\nu -3}{2}}}{2^{\nu -1}}e^{\frac{i\pi (2-\nu )}{2}}\Gamma \left(\frac{3-\nu }{2}\right)$$

(A20) $$\left[{\left(\rho -i0\right)}^{-\frac{1}{2}}{\left(\rho +i0\right)}^{\frac{\nu -3}{2}}+e^{i\pi (\nu -2)}{\left(\rho +i0\right)}^{-\frac{1}{2}}{\left(\rho -i0\right)}^{\frac{\nu -3}{2}}\right].$$

Simplifying terms we obtain

(A21) $$h\left(\rho ,\nu \right)=\frac{\pi^{\frac{\nu -3}{2}}}{2^{\nu -1}}e^{\frac{i\pi (2-\nu )}{2}}\Gamma \left(\frac{3-\nu }{2}\right)\left[\rho_{+}^{\frac{\nu -4}{2}}+e^{\frac{i\pi (\nu -2)}{2}}\rho_{-}^{\frac{\nu -4}{2}}\right].$$

Thus, in four dimensions

(A22) $$h_{4}\left(\rho \right)=\delta \left(\rho \right)*\delta \left(\rho \right)=\frac{\pi }{2}Sgn\left(\rho \right).$$

Note that this convolution does not make sense in a four-dimensional Euclidean space, since in that case δ(ρ)≡0.

As a second example we calculate the convolution $\delta \left(\rho -m^{2}\right)*\delta \left(\rho -m^{2}\right)$. In this instance we have

$$h\left(\rho ,\nu \right)=\frac{\pi^{\frac{\nu -3}{2}}}{2^{\nu -1}}e^{\frac{i\pi (2-\nu )}{2}}\Gamma \left(\frac{3-\nu }{2}\right)$$

(A23) $$\left[{\left(\rho -i0\right)}^{-\frac{1}{2}}{\left(\rho -2m^{2}+i0\right)}^{\frac{\nu -3}{2}}+e^{i\pi (\nu -2)}{\left(\rho +i0\right)}^{-\frac{1}{2}}{\left(\rho -2m^{2}-i0\right)}^{\frac{\nu -3}{2}}\right].$$

When ν=4 we find

(A24) $$\delta \left(\rho -m^{2}\right)*\delta \left(\rho -m^{2}\right)=\;\frac{\pi }{4}\left[{\left(\rho -i0\right)}^{-\frac{1}{2}}{\left(\rho -2m^{2}+i0\right)}^{\frac{1}{2}}+e^{i\pi (\nu -2)}{\left(\rho +i0\right)}^{-\frac{1}{2}}{\left(\rho -2m^{2}-i0\right)}^{\frac{1}{2}}\right].$$

## References

[1] Lynden-Bell D., Lynden-Bell R.M. (1977). On the negative specific heat paradox. Mon. Not. R. Astron. Soc..

[2] Padmanabhan T. (1990). Statistical mechanics of gravitating systems. Phys. Rep..

[3] Padmanabhan T., Dauxois T., Ruffo S., Arimondo E., Wilkens M. (2002). Statistical Mechanics of gravitating systems in static and cosmological backgrounds. Dynamics and Thermodynamics of Systems with Long-Range Interactions.

[4] Padmanabhan T. (2000). Theoretical Astrophysics: Volume 1, Astrophysical Processes.

[5] Binney J., Tremaine S. (1987). Galactic Dynamics.

[6] Bollini C.G., Giambiagi J.J. (1972). Lowest order “divergent” graps in *ν*-dimensional space. Phys. Lett. B.

[7] Bollini C.G., Giambiagi J.J. (1972). Dimensional Renorinalization: The Number of Dimensions as a Regularizing Parameter. Nuovo Cim. B.

[8] Bollini C.G., Giambiagi J.J. (1996). Dimensional Regularization in Configuration Space. Phys. Rev. D.

[9] Bietenholz W., Prado L. (2014). Revolutionary physics in reactionary Argentina. Phys. Today.

[10] Plastino A., Rocca M.C. (2018). Quantum Field Theory, Feynman and Wheeler Propagators, Dimensional Regularization in Configuration Space and Convolution of Laurent Invariant Distributions. J. Phys. Commun..

[11] Zamora J.D., Rocca M.C., Plastino A., Ferri G.L. (2018). Application of a dimensionally regularized Tsallis’ Statistical Mechanics to Newton’s gravitation. Physica A.

[12] Rocca M.C., Plastino A., Ferri G.L., Zamora D. (2018). Dimensionally regularized Boltzmann-Gibbs Statistical Mechanics and two-body Newton’s gravitation. Physica A.

[13] Tsallis C. (2009). Introduction to Nonextensive Statistical Mechanics Approaching a Complex World.

[14] Gradshteyn I.S., Rizhik I.M. (1965). Table of Integrals Series and Products.

[15] Erdelyi A. (1954). Tables of Integral Tranforms.

[16] Thirring W. (1970). Systems with negative specific heat. Zeitschrift für Physik A Hadrons and Nuclei.

[17] Thirring W. (2006). The Story of Negative Specific Heat.

[18] Verlinde E. (2011). On the Origin of Gravity and the Laws of Newton. J. High Energy Phys..

[19] Bollini C.G., Escobar T., Rocca M.C. (1999). Convolution of Ultradistributions and Field Theory. Int. J. Theor. Phys..

[20] Bollini C.G., Rocca M.C. (2004). Convolution of Lorentz Invariant Ultradistributions and Field Theory. Int. J. Theor. Phys..

[21] Bollini C.G., Rocca M.C. (2004). Convolution of Even Ultradistribution and Field Theory. Int. J. Theor. Phys..

[22] Bollini C.G., Marchiano P., Rocca M.C. (2007). Convolution of Ultradistributions, Field Theory, Lorentz Invariance and Resonances. Int. J. Theor. Phys..

[23] Silva J.S.E. (1958). Les fonctions analytiques comme ultra-distributions dans le calcul opérationnel. Math. Ann..

[24] Schwartz L. (1966). Théorie des Distributions.

[25] Gel’fand I.M., Shilov G.E. (1964). Generalized Functions.

